# Neutrophil-to-lymphocyte ratio, Factor VIII and Antithrombin III: inflammatory-clotting biomarkers in glioma

**DOI:** 10.17179/excli2021-3831

**Published:** 2021-07-08

**Authors:** Tatiana Koudriavtseva, Veronica Villani, Svetlana Lorenzano, Diana Giannarelli, Enea Gino Di Domenico, Annunziata Stefanile, Marta Maschio, Giovanna D'Agosto, Fulvia Pimpinelli, Antonio Tanzilli, Edvina Galiè, Andrea Pace

**Affiliations:** 1 Department of Clinical Experimental Oncology, IRCCS Regina Elena National Cancer Institute, IFO, Via Elio Chianesi 53, 00144, Rome, Italy; 2 Department of Human Neurosciences, Sapienza University of Rome, Rome, Italy; 3 Biostatistics, IRCCS Regina Elena National Cancer Institute, IFO, Rome, Italy; 4 Clinical Pathology and Microbiology Unit, IRCCS San Gallicano Institute, IFO, Rome, Italy

**Keywords:** biomarker, glioblastoma, glioma, neutrophil-to-lymphocyte ratio, Antithrombin III, Factor VIII, inflammation, coagulation

## Abstract

One of the key difficulties in glioma treatment is our limited ability to consistently assess cancer response or progression either by neuroimaging or specific blood biomarkers. An ideal biomarker could be measured through non-invasive methods such as blood-based biomarkers, aiding both early diagnosis and monitoring disease evolution. This is a single-center, case-control, 10-year retrospective, longitudinal study. We evaluated routine coagulation factors in 138 glioma patients (45 Females/93 Males; median [range] age, 56.4 [27-82] years; 64 non-recurrent/74 recurrent) and, for comparison, in 56 relapsing-remitting MS patients (41 Females/15 Males; 40.8 [25-62] years, 35 stable/21 active) and 23 controls (16 Females/7 Males; 41.7 [24-62] years) as well as Neutrophil-to-lymphocyte ratio (NLR) in subgroups of 127 glioma patients, 33 MS patients and 23 healthy controls. Secondly, we assessed whether these indicators could be predictive of overall (OS) and progression-free survival (PFS) in glioma patients. NLR, d-dimer, Antithrombin III and Factor VIII were significantly higher in glioma patients compared to both MS patients and controls (p<0.0001 for all). ROC curves confirmed that either NLR, Antithrombin III or Factor VIII were moderately accurate biomarkers (0.7<AUC<0.9) for glioma patients compared to other two groups whereas d-dimer was a moderately accurate marker for glioma only when compared to controls. In multivariable analysis, NLR ≥ 4.3 (median) (HR 1.53 [95 % CI 1.04-2.26], p=0.03) together with the Karnofsky Performance Status (KPS) ≥ 80 (median) (0.46 [0.31-0.69], p<0.0001) and use of steroids (1.75 [1.19-2.57], p=0.004) resulted independent predictors of OS while only KPS was independently associated with PFS. Our study showed increased levels of either NLR, Antithrombin III, Factor VIII, or d-dimer in glioma patients compared to MS patients and controls, where the first three represented moderately accurate biomarkers for this cancer. Among these markers, only NLR was found to be predictive for OS along with severe disability and steroid therapy.

## Introduction

The incidence of central nervous system (CNS) cancers increased between 1990 and 2016 leading to substantial morbidity and mortality rates worldwide, seeing countries like China, the USA, and India with the highest number of incident cases (GBD 2016 Brain and Other CNS Cancer Collaborators, 2019[14]). The prevalence of CNS glial tumors in Europe estimated by the RARECARE working group was around 30 per 100,000 (Crocetti et al., 2012[6]). There were more than 154,000 people in the EU suffering from CNS glial and non-glial cancers at the beginning of 2008, representing a very heterogeneous group in terms of frequency, prognosis, and treatment (Crocetti et al., 2012[6]). 

Gliomas, which are tumors of glial cell origin, represent the most common histological type of cancers with about 81 % of primary malignant brain cancers including high-grade glioma (HGG) (anaplastic astrocytoma, anaplastic oligodendroglioma, glioblastoma (GBM)) and low-grade gliomas (astrocytoma, oligodendroglioma) (Ostrom et al., 2014[45]). GBM is the most frequent (∼45 % of all gliomas) and aggressive form of gliomas with an incidence of 3.19 cases per 100,000 persona year, and has a very poor prognosis showing a 2-year survival rate ranging from 26 % to 33 % and a 5-year and 10-year survival rate of only 4-5 % and 0.71 %, respectively (Batash et al., 2017[4]; Tykocki and Eltayeb, 2018[60]). Specific biomarkers recently discovered by genomic analyses of glioma include O⁶-methylguanine-DNA methyltransferase methylation (*MGMT*), isocitrate dehydrogenase (*IDH1*) mutation, and a glioma cytosine-phosphate-guanine island methylator phenotype, all indicating improved patient survival (Ostrom et al., 2014[45]). Population statistics have shown no notable improvement in survival rates for patients with GBM in the last three decades in spite of a significant improvement in the ability to diagnose and treat brain tumors. 

On the other hand, the diagnosis of glioma is not always a forthright process because many other neurological diseases could mimic brain cancers on neuroimaging and even on histological examination due to sampling errors or misinterpretation of pathological tissue features (Omuro et al., 2006[44]). The differential diagnosis of glioma firstly includes multiple sclerosis (MS), an inflammatory-demyelinating and neurodegenerative disease of the CNS affecting prevalently young women (Yadav et al., 2015[67]). Furthermore, the diagnosis can be complicated by several types of brain tumors that have been described in association with MS without having a clear understanding on whether their occurrence is accidental or a consequence to causal events (Plantone et al., 2015[50]).

In several types of neoplasia, the inflammatory microenvironment is a pre-existing condition promoting a malignant change (Mantovani et al., 2008[38]). In addition, the subsequent induced inflammation in the tumor microenvironment further promotes the proliferation and survival of cancer cells and can stimulate angiogenesis and the development of metastases. In these conditions, the adaptive immune reaction is subverted with altering responses to hormones and chemotherapeutic agents. There is growing evidence indicating that abnormalities of coagulation pathways are involved in the pathogenesis of several neurological diseases (Krenzlin et al., 2016[30]; Plantone et al., 2018[49]). The CNS is the only site of extra-hepatic thrombin expression that seems to have a role in brain development, defense, and regeneration (Dihanich et al., 1991[8]). In fact, either brain cancer or MS are associated with vascular and thrombotic events, in particular the association with venous thromboembolism (VTE) is common in glioma reaching up to 30 % of patients with HGG (Jo et al., 2014[24]; Koudriavtseva, 2014[27]). The risk of VTE in brain cancer could be related to the production of tissue factor, a potent procoagulant. Its upregulation influences both the coagulation pathway and oncogenic signaling mechanisms consequently leading to cancer progression (Jo et al., 2014[24]). A detrimental effect of thrombin in GBM by enabling tumor cell seeding and metastasis was also demonstrated, resulting in increased tumor cell growth and angiogenesis (Krenzlin et al., 2017[29]). Preclinical studies showed an inhibitory effect of heparins on GBM growth, cell invasion and angiogenesis (Schnoor et al., 2015[54]). 

This is not surprising since innate immunity with its clotting components is involved in any inflammatory, degenerative, infectious, traumatic, and neoplastic conditions (Engelmann and Massberg, 2013[9]). Although acute inflammation is crucial for survival during health-damaging events, systemic chronic inflammation mainly reflecting the action of innate immunity could lead to several inflammation-related diseases such as autoimmune and neurodegenerative disorders, and cancer (Furman et al., 2019[12]). Some coagulation factors or innate immune cells such as neutrophils and their relationship to adaptive immune cells - lymphocytes - (neutrophil to lymphocyte ratio, NLR) are currently considered markers of systemic inflammation in neoplastic, inflammatory, or infective diseases (Sacdalan et al., 2018[52]; Subramaniam and Scharrer, 2018[58]; Pierscianek et al., 2019[47]). Several inflammatory markers (C-reactive protein, interleukin-6 tumor necrosis factor alpha, sialic acid) and coagulation markers (prothrombin fragments 1+2, endogen thrombin generation, vascular endothelial growth factor (VEGF) and soluble VEGF-receptor 1) have been found at elevated levels in patients with GBM but no associations between these markers and overall survival (OS) or progression-free survival (PFS) were found (Reynés et al., 2011[51]).

Indeed, one of the key difficulties in glioma treatment is our limited ability to consistently assess cancer response or progression either by both neuro-imaging or the specific blood biomarkers (Holdhoff et al., 2013[21]). An ideal biomarker could be measured through non-invasive methods such as blood-based biomarkers, helping both early diagnosis and grading accuracy, and monitoring disease evolution and therapy response (Linhares et al., 2020[33]). Currently, no serum screening or clinical follow-up biomarkers are available for glioma due to their low incidence and their apparent ability to develop *de novo* within a few weeks or months. However, several inflammatory markers and coagulation factors have been found to be associated with HGG. Therefore, there is a great need for biomarkers in glioma, which should be sufficiently sensitive and specific, easy to use in routine clinical practices, and predictive of both PFS and OS.

Given the above, coagulation factors could be an optimal candidate, D-dimer was the most frequently used. It is a fibrinolysis-specific degradation product, and its levels indicate both balance of intravascular-extravascular fibrin formation and its clearance (Greenberg, 2017[16]). Elevated d-dimer levels reflect the persistent activation of clotting-inflammatory pathways. Overall, d-dimer was found to be increased in many diseases, particularly during their acute/active phase (e.g., cerebral vascular incidents, traumatic brain injury, and brain cancer) (Wiseman et al., 2014[65]; Zhang et al., 2018[70]; Navone et al., 2019[42]). Recent studies on all-type cancers have mainly evaluated the association between d-dimer and VTE providing partially contrasting results (Misch et al., 2013[41]; Thaler et al., 2014[59]; Streiff et al., 2015[57]; Pace et al., 2018[46]). However, only a few studies investigated the prognostic value of increased d-dimer levels regardless of the VTE (Hoke et al., 2011[20]; Ay et al., 2012[2]; Halaby et al., 2015[17]). Conversely, in some previous studies the NLR and platelets-to-lymphocytes ratio (PLR) has been demonstrated to be the prognostic factor for both OS and PFS in patients with GBM (Kaya et al., 2017[25]; Bao et al., 2018[3]; Wang et al., 2019[62]; Yang et al., 2019[68]). 

The main purpose of this 10-year retrospective study was to identify possible biomarkers in glioma comparing some routine coagulation factors such as d-dimer, fibrinogen, Antithrombin III (ATIII), Factor VIII (FVIII), PT, PTT and the ratio of complete blood count components such as NLR and PLR among the following subgroups: a) patients with glioma, patients with MS, and controls; b) patients with non-recurrent and patients with recurrent glioma. As a secondary objective, we evaluated the predictability of these potential biomarkers for PFS and OS in glioma patients.

## Materials and Methods

### Study design

This is a single-center, case-control, retrospective, longitudinal study. Demographic, clinical and laboratory data of glioma and MS patients who had at least one d-dimer measurement during the 10-year period (from 2009 to 2019) were obtained from our longitudinal databases. Most of the participants also had blood count and several clotting factors measured at the same time of d-dimer. Patients with VTE and/or on heparin therapy were excluded. Controls were subjects without known diseases with at least one d-dimer measurement included in our database. It was not possible to match patients to controls since only a limited number of controls were found.

### Clinical and laboratory evaluation

Functional patient status was assessed by Karnofsky Performance Status (KPS) in glioma and by Expanded Disability Status Score (EDSS) in MS. Clinical worsening, progression, and recurrence in glioma patients were defined by the Response Assessment in Neuro-Oncology (RANO) criteria (Wen et al., 2010[63]). Glioma patients' PSF and OS were considered starting with the date of collecting blood for d-dimer levels.

All coagulation parameters, including d-dimer, were assayed by clotting, chromogenic, and immunological methods on a fully-automated ACLTOP analyzer using HemosIL® commercial kits. Complete blood count was performed by the Beckman Coulter's Automated Intelligent Morphology flow cytometric technology, Digital Signal processing, Multi-Transducer Module and advanced algorithms. 

All laboratory markers investigated by the technical procedures used in this study are already well established and are part of the clinical laboratory practice. For each parameter of the blood count performed with the Beckman Coulter's Automated Intelligent Morphology flow cytometric technology, three measurements are performed as set in the analyzer. The quality controls that are routinely performed analyze three levels (high, medium, low) for each analyte and must respond to the expected mean and standard deviation results reported for each lot in accordance to the company's indications.

### Statistical analysis

Descriptive statistics were used to describe patient characteristics. Median values were used as cut-off points for subgroup analyses because most parameters used did not have a normal distribution in the three different groups. Hence, the series was divided into two parts with the same sample size and were not biased by the selection of the best cut-offs determined based on several criteria. Correlation among quantitative variables was tested with the Spearman's coefficient. The association between variables was tested by the Pearson chi-square or Fisher's Exact test. The comparison between groups was performed by the Mann-Whitney U test or Kruskal-Wallis non-parametric tests. A log-rank test was adopted to assess differences between subgroups. The Kaplan-Meier method served to estimate PFS and OS. The Hazard Ratio (HR) and the 95 % confidence intervals (95 % CI) were calculated for each variable by the Cox regression model. Multivariable analysis was performed using a forward stepwise approach with the enter and remove limit set at 0.05 and 0.10, respectively. The Area Under Curve (AUC) was calculated with the ROC (Receiver Operating Characteristic) analysis to assess biomarker performances in terms of discrimination between groups. ROC curves and AUC were reported and cut-off values were identified according to the Youden statistics. The level of significance was set at p≤0.05.

The IBM-SPSS (21.0) licensed statistical programs were used for all analyses. 

## Results

### Participant's demographic and clinical characteristics

Overall, 138 glioma patients (Grade 2-3, n=41; Grade 4, n=97), 56 MS patients and 23 controls were included in the study. Patient demographic and clinical characteristics are shown in Table 1(Tab. 1). Age correlated with both disease duration (rho=-0.47, p<0.0001) and KPS (rho=-0.21, p=0.01) in glioma patients, and with either disease duration (rho=0.32, p=0.02) or EDSS (rho=0.34, p=0.01) in MS patients. There was a significant difference in either age, disease duration or KPS between patients with glioma grade 4 (they were older with shorter disease duration, and lower KPS) and grade 2-3 (Table 1(Tab. 1)). Glioma patients with grade 4 showed a significantly smaller percentage of *IDH1* mutation than those with grade 2-3 (Table 1(Tab. 1)).

The median PFS and OS were 12.4 months (CI 95 % 10.5-14.3) and 23.5 months (CI 95 % 20.3-26.7), respectively. Neither PFS nor OS in Grade 2-3 glioma patients differed from those patients with Grade 4 glioma. For this reason, we decided to consider all glioma patients in an overall group: one-year OS was observed in 80.3 % of patients, 2-year OS in 47.3 %, and 10-year OS only in 9.1 % of cases.

### Correlations between age and laboratory parameters 

Age of all participants was correlated with: neutrophils number (rho=0.335, p<0.0001), NLR (rho=0.320, p<0.0001), d-dimer (rho=0.232, p=0.001), aPTT (rho=-0.198, p=0.012), FVIII (rho=0.209, p=0.039), fibrinogen (rho=0.152, p=0.05).

### Correlations between d-dimer, NLR and other laboratory parameters

D-dimer correlated with either NLR (rho=0.39, p<0.0001), neutrophil count (rho=0.33, p<0.0001) or lymphocyte count (rho=-0.23, p=0.002) in all study participants. Positive correlations were found between d-dimer and either fibrinogen (p<0.0001), ATIII (p<0.0001), FVIII (p<0.0001), white blood cells (WBC) (p=0.001); negative correlations were observed between d-dimer and either mean platelet volume (MPV) (p=0.01), Hb (p=0.02), or aPTT (p=0.002). There were no correlations between d-dimer and either thrombocyte count (p=0.20), PT (p=0.51), PTT (p=0.97), or platelet distribution width (PDW) (p=0.30).

### Associations between demographic/clinical patient characteristics and laboratory parameters 

D-dimer results differed significantly across the three groups where higher levels were found in glioma patients; p values were <0.0001 for the comparisons performed among glioma, MS and controls; glioma vs. MS; glioma vs. controls and equals to 0.02 for the comparison MS vs. controls (p=0.02). Similarly, NLR, ATIII and FVIII resulted significantly different among the three groups with higher levels resulting in glioma patients (Table 2(Tab. 2)).

#### Glioma patients 

There were no differences in laboratory parameters between males and females, except for d-dimer for which a trend towards statistical significance was observed for women (female/male, median [range], 220 [28-3665] ng/ml vs 139 [3-3193] ng/ml, p=0.09), as well as between patients within a lower age bracket than the median value of 56 years and those aged ≥ 56 years, and between patients with disease duration shorter than the median value of 10 months and those with disease duration ≥ 10 months. Patients with KPS ≥ median of 80 (cutoff indicating a relatively conserved functional status) compared to patients with KPS < 80 had significantly lower d-dimer levels (121 [3-1653] ng/ml vs 210 [21-3665] ng/mL, p=0.004) with a trend toward a lower ATIII (119 % [79-160] vs 127 % [92-181], p=0.07). There were no significant differences for all the laboratory parameters between patients with and without recurrent glioma and between patients with or without *MGMT *methylation or *IDH1* mutation. 

Glioma patients with d-dimer levels ≥ median values of 180 ng/ml differed from those with d-dimer levels < 180 ng/ml in age (58.8±12.8 vs 52.8±13.7 years, p=0.009), gender (F/M 28/40 [70 %] vs 17/53 [32.1 %], p=0.03), KPS (77.2±16.7 vs 87±13.1, p=0.001). D-dimer levels were not different when compared between patients treated or not with steroids (median [range], 182 [33-3665] ng/ml vs 179 [3-3193] ng/ml, p=0.46). Glioma patients with NLR ≥ median values of 4.3 did not differ from those with levels <4.3. NLR was not different between patients treated or not with steroids (median [range], 4.2 [1-44] vs 4.5 [1-18.8] respectively, p=0.86). 

There was a trend toward the statistical significance for the correlation between d-dimer levels and NLR (rho=0.16, p=0.07). 

**Figure 1:**
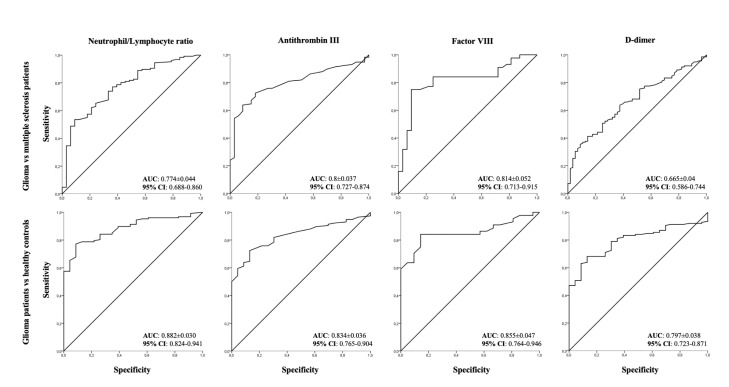
ROC curve analysis of Neutrophyl/Lymphocyte rate and coagulation factors (Antithrombin III, Factor VIII, d-dimer) in glioma patients versus multiple sclerosis patients or healthy controls

ROC curves confirmed that NLR, ATIII and FVIII were moderately accurate biomarkers for glioma compared to both MS patients and healthy controls whereas d-dimer was a moderately accurate marker once compared to controls but not to MS patients (Table 3(Tab. 3), Figure 1(Fig. 1)). 

#### MS patients

MS patients with d-dimer ≥ median levels of 105 ng/ml did not differ in age, gender, EDSS, disease duration from those with d-dimer levels < 105 ng/ml as patients with NLR ≥ median value of 2.2 were not different from those with NLR < 2.2. Patients in relapse compared to remission had a significantly higher d-dimer (median [range] 165 [39-622] ng/ml vs 85 [22-265] ng/ml, p=0.009) and ATIII (112 % [107-136] vs 106 % [85-119], p=0.02). In 33 patients with available NLR value was not significantly higher in subjects with relapse compared with those in remission (2.5 [1-20] vs 1.98 [0.5-5.8], p=0.3). There was a trend toward a higher NLR in MS patients compared with controls (p=0.08). D-dimer levels correlated with either NLR (rho=0.54, p=0.001), neutrophils (rho=0.38, p=0.03) or lymphocytes (rho=-0.37, p=0.04). ROC curves did not show accurate biomarkers for MS patients compared to healthy controls, likely in part due to the small numbers (Table 3(Tab. 3)).

### Predictive laboratory parameters for PFS and OS in glioma patients

For the predictive laboratory parameters for PFS and OS in glioma patients see Table 4(Tab. 4). In the univariate analysis, a lower KPS (≥ 80 vs < 80, HR 0.39 [95 % CI 0.27-0.56], p<0.0001), steroid therapy (HR 1.54 [95 %CI 1.07-2.20], p=0.02), the absence *IDH1* mutation (*IDH1*-wild-type) (HR 3.66 [95 % CI 1.12-12.0], p=0.03) were significantly associated with a shorter PFS. The median PFS for patients with KPS ≥ 80 and < 80 was 7 months (95 %CI: 4.8-9.2) and 3 months (95 % CI: 2.4-3.6), respectively (p<0.0001); for patients with or without steroid therapy, 5 months (95 % CI: 3.8-6.2) and 7 months (95 % CI: 3.2-10.7), respectively (p=0.02); for patients with or without *IDH1 *mutation, 18 months (95 % CI: NE) and 6 months (95 % CI: 3.3-8.7), respectively (p=0.03). Multivariable analysis selected KPS as the only independent predictive factor of PFS (0.39 [95 % CI 0.27-0.56], p<0.0001).

**Figure 2:**
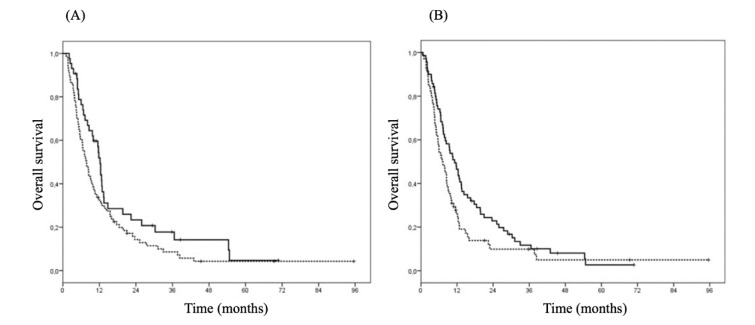
Overall survival by d-dimer and Neutrophil/Lymphocyte ratio (NLR). A. Overall survival by d-dimer (straight line: d-dimer < 180; dotted line: d-dimer ≥ 180) (HR 1.45; 95 % CI 1.02-2.07, p=0.04) in glioma patients. B. Overall survival by NLR (straight line NLR < 4.3; dotted line NLR ≥ 4.3) (HR 1.44, 95 % CI 0.97-2.13, p=0.07) in glioma patients

Similarly, in the univariate analysis a lower KPS (≥ 80 vs < 80, HR 0.34 [95 % CI 0.23-0.50], p<0.0001), high levels of d-dimer (≥ 180 vs < 180, HR 1.45 [95 % CI 1.02-2.07], p=0.04) (Figure 2A(Fig. 2)), steroid therapy (1.89 [95 % CI 1.31-2.72] p=0.001), and the absence of *MGMT* methylation (1.60 [1.01-2.52], p=0.046) were significantly associated with a shorter OS with a trend toward a statistical significance for high NLR values (≥ 4.3 vs < 4.3, HR 1.44 [95 % CI 0.97-2.13], p=0.07) (Figure 2B(Fig. 2)) and for the absence of the *IDH1* mutation (3.08 [0.94-10.14], p=0.06). The median OS for patients with KPS ≥ 80 and < 80 was 12.4 months (95 % CI: 9.2-15.6) and 5.6 months (95 % CI: 4.0-7.2), respectively; for patients with d-dimer ≥ 180 and < 180, 7.1 months (95 % CI: 4.8-9.3) and 11.2 months (95 % CI: 8.3-14.1), respectively; for patients with and without steroids, 7.4 months (95 % CI: 5.7-9.1) and 12.4 months (95 % CI: 8.3-16.5), respectively; for patients with and without *IDH1* mutation 34.4 months (95 % CI: 0-107) and 10.2 months (95 % CI: 7.2-13.2); for patients with and without *MGMT*-methylation 12.7 months (95 % CI: 8.0-17.4) and 9.8 months (95 % CI: 8.3-11.3). 

In the multivariable analysis of KPS (≥ 80 vs < 80, HR 0.46 [95 % CI 0.31-0.69], p<0.0001), high NLR values (≥ 4.3 vs < 4.3, HR 1.53 [95 % CI 1.04-2.26], p=0.03), and steroid therapy (1.75 [1.19-2.57]; p=0.004) resulted independent predictors of OS. However, the ROC curve showed that NLR was not an accurate biomarker for OS (AUC 0.62, SD 0.076; 0.47-0.77, threshold value of 7).

*MGMT*-methylation, *IDH1 *mutation and FVIII were not included in the multivariate analysis as they were not available in all patients.

See also the Supplementary data.

## Discussion

Our study found that some coagulation factors such as d-dimer, ATIII and FVIII, as well as NLR were significantly different among the three study populations: glioma patients, MS patients, and healthy controls. In particular, d-dimer levels were higher in glioma patients compared to MS patients, which in turn were higher than in the control group, and even in MS relapse compared to remission, therefore, growing from a healthy to inflammatory status (or from less to more inflammation) up to neoplastic disease. NLR was significantly increased in glioma patients compared to MS patients and showed a trend toward a statistical significance for higher values in MS patients than in the control group. Similarly, both ATIII and FVIII were increased in glioma patients compared to either MS patients or healthy controls, with ATIII being higher in MS relapse compared to remission. All these factors were significantly correlated with each other as well as with age (except for ATIII). These correlations could be driven by possible biases in our study since glioma patients were older than other group participants. Nevertheless, some age-related coagulation factors such as PTT and fibrinogen did not differ between the three groups in our study. Indeed, FVIII and fibrinogen are acute phase reactants of inflammatory-thrombotic processes which gradually increase with aging in the normal general population along with the development of acquired age-related prothrombotic risk factors such as cancer and autoimmune disorders that enhance the thrombotic risk in these subjects (Favaloro et al., 2014[10]). Conversely, for the natural anticoagulants such as protein C, protein S, and ATIII age-related changes have not been found in the general population.

Moreover, we found NLR to be an independent predictor of a shorter OS in the multivariable analysis although it did not result to be a sufficiently accurate biomarker. A higher d-dimer was associated with OS only in the univariate but not in the multivariable analysis. It was significantly higher in patients with a lower KPS (i.e. with a greater functional disability), which in turn was predictive of both PFS and OS. It is important to underline that the predictive value of NLR was independent of steroid therapy even though steroids normally increase the number of circulating neutrophils and consequently NLR. In our patients, the values of both NLR and d-dimer did not differ between the groups of patients under steroid therapy possibly reflecting the influence of many other clinical conditions.

Furthermore, steroid therapy resulted to be a negative predictive marker of OS in the multivariable analysis and it was also associated with PFS in the univariate analysis. Our results confirmed several previous studies and reviews which supported that steroid administration reduces OS and PFS in glioma patients highlighting the need for its restricted use in these cancers (Shields et al., 2015[56]; Wong et al., 2015[66]; Pitter et al., 2016[48]; Ly and Wen, 2017[35]; Jessurun et al., 2019[23]). Steroid therapy with dexamethasone reduces cerebral edema inducing cytokine cascades with a global immunosuppression that interfere with immune functions necessary for the treatment of GBM.

Similarly, our findings regarding NLR are in line with the results of several recently published studies. Kaya and colleagues found that OS significantly correlated with a systemic inflammatory response (i.e., NLR ≥ 5 and/or PLR ≥ 150) and NLR before treatment has been demonstrated to be a prognostic factor in patients with GBM (Kaya et al., 2017[25]). A preoperative NLR of less than 3.2 was found to be associated with a better outcome in patients with Grade 2-4 glioma (Wang et al., 2019[62]). Interestingly, *IDH*-mutant glioma seems to contain a higher lymphocyte infiltration than *IDH*-wt glioma, and the presence of neutrophils or lymphocytes was found to be an independent prognostic factor for this tumor (Wang et al., 2019[62]). Yang et al. revealed the cutoff of NLR ≥ 2.8 as an independent risk factor for a decreased OS together with age ≥ 50 years in HGG patients with gross total resection (Yang et al., 2019[68]). In the multivariable models, NLR with acut-off of 3 resulted to have a prognostic value for OS, together with tumor grade, extent of resection, adjuvant radiotherapy/temozolomide, also in elderly patients (age ≥ 65 years) with HGG (Gan et al., 2019[13]). Weng et al found that NLR ≥ 4.0, along with age ≥ 60, KPS ≤ 70, incomplete tumor resection, incomplete Stupp protocol completion, and the *IDH1*-wt, independently predicted worse outcome (Weng et al., 2018[64]). Similarly, NLR > 4 resulted as the only independent predictor of OS in the multivariable model including PLR (> 200), tumor size (≥ 5 cm), WHO grade (III/IV), and KPS (< 70) (Wang et al., 2018[61]). Another study demonstrated that patients with either a high compared to low NLR, PLR or monocyte-to-lymphocyte ratio (MLR) had shorter OS (Bao et al., 2018[3]). 

Even temporary changes in NLR, for example its decrease during radiotherapy/temozolomide treatment, were associated with longer OS in GBM, and were confirmed as independent predictors of OS together with age, total temozolomide cycles, and performance status (Mason et al., 2017[40]). However, some studies did not confirm the association between a higher NRL and shorter survival in GMB, except in the subgroup that completed the Stupp protocol (Lopes et al., 2018[34]). A recent systematic review concluded that no blood-based biomarker for HGG achieved level I evidence, whereas only ten predictive biomarkers, which included NLR, reached level II evidence (Pierscianek et al., 2019[47]).

At the same time, hematological indices such as NLR and monocytes/lymphocytes ratio (MLR) have been used as inflammatory biomarkers also in other disorders including MS. NLR was found to be significantly higher in MS patients than in healthy controls, especially in the relapse compared to remission phase (Demirci et al., 2016[7]; Al-Hussain et al., 2017[1]; Bisgaard et al., 2017[5]). A large study based on the data from the Danish Multiple Sclerosis Biobank confirmed that MS patients in the early phase compared with healthy controls had a significantly higher NLR, weakly correlated with an MS severity score (Hasselbalch et al., 2018[18]). The cut-off values of NLR identified to predict MS diagnosis and activity were 2.04 and 3.9, respectively. Furthermore, a high NLR was found to be an independent predictor of disability progression (Demirci et al., 2016[7]). In our study, NLR showed a trend towards a significant difference between MS patients and controls, but not between relapsing and remitting patients, most likely due to the overall small number of participants having NLR. Nevertheless, there are controversial results regarding the correlations between NLR and EDSS score or MS form (Bisgaard et al., 2017[5]; Hemond et al., 2019[19]). 

Regarding the role of d-dimer in outcome prediction for glioma patients, only few studies evaluated this aspect even though elevated d-dimer was associated with an increased risk of mortality in a variety of cancers independent of age, sex, documented VTE, and types of malignancy (Knowlson et al., 2010[26]; Ay et al., 2012[2]). In a comprehensive meta-analysis of 49 studies, increased pretreatment plasma d-dimer levels in solid tumors was markedly associated with poor OS and shorter PFS (Li et al., 2018[32]). In 23 GBM patients, d-dimer levels obtained two-three weeks after surgery or needle biopsy but prior to chemotherapy or radiotherapy, were significantly associated with both mortality and PFS, even after a short 7.3-month follow-up (Hoke et al., 2011[20]). Sciacca et al. reported that in patients with HGG, the higher incidence of VTE compared with both healthy controls and MS patients, could not be explained by genetic risk factors but by intrinsic cancer determinants including higher plasma levels of coagulation factors such as d-dimer (Sciacca et al., 2004[55]). Navone et al. showed that GBM patients compared to those with meningioma had reduced PT and aPTT, as well as high levels of both d-dimer and von Willebrand factor (vWF) (Navone et al., 2019[42]). This condition is defined as a hypercoagulable profile and is associated with reduced OS. Likewise, increased levels of either d-dimer, antiphospholipid antibodies or other coagulation factors were found in the CNS inflammatory-demyelinating diseases, especially in relapse, and was associated with a worse outcome (Iong et al., 2013[22]; Koudriavtseva et al., 2014[28]).

One new finding of our study is the demonstration that some routine coagulation factor such as ATIII and FVIII could be the moderately accurate biomarkers for glioma patients. Increased FVIII is a known independent risk factor for thrombosis, which could be found during numerous pathologies including chronic inflammation and malignancy. The relationship between high FVIII levels and risk of all-cause mortality was shown not only in patients with venous thrombosis but also in people from the general population in the large-cohort of the MEGA follow-up study (Yap et al., 2015[69]). The association between FVIII and mortality may be in part explained by several underlying factors such as chronic comorbidities and chronic inflammation. 

Activated FVIII works as a cofactor for Factor IXa while in its unactivated form FVIII circulates in plasma in a complex with vWF, which is a FVIII-related antigen that mediates platelet adhesion to endothelial and subendothelial surfaces (Saenko et al., 1999[53]). Usually, there is a concurrent increase of FVIII and vWF: Ag levels.

The morphological examination and the computerized morphometric analysis showed groups with vWF-FVIII positive glial neoplastic cells to be significantly higher only in GBM and not in astrocytoma II compared to background, suggesting that the activity of vWF-FVIII within these cells is linked to the malignancy of gliomas as well as their location near the vascular glomeruloid structures associated with endothelial cells (Nowacki and Tabaka, 2003[43]).

Again, the preoperative antigen plasma median levels of vWF:Ag were significantly higher in GBM than in meningiomas and increased vWF levels were associated with a threefold higher risk of death in GBM patients (Marfia et al., 2016[39]). Even in lower grade gliomas, vWF gene expression had a negative prognostic effect as it emerged from the genomic data of The Cancer Genome Atlas (Lehrer et al., 2018[31]). There was a significant relationship between growing staining intensity of vWF in microvessels and increasing grade of glioma confirming that the most important pathologic criteria for the diagnosis of HGG is microvessel proliferation (Mahzouni et al., 2010[37]). To the best of our knowledge, there are no studies reporting on the prognostic role of ATIII in glioma except for one paper that examined its predictive value in patients with GBM under treatment with perillyl alcohol which has antiangiogenic and anti-tumoral properties (Fischer et al., 2008[11]). After a four-month therapy, ATIII and fibrinogen were respectively down- and up-regulated as GBM advanced with a possible link between tumor progression and ATIII expression level. Thus, this study supported an association between reduced levels of ATIII for cancer evolution. 

ATIII is an important endogenous anticoagulant protein acting through the inactivation of either thrombin, Factor Xa or other enzymes in the intrinsic coagulation pathway and in this manner decreasing fibrin formation. It is important to underline that thrombin acts on the CNS in a dosage-dependent manner (Gofrit and Shavit-Stein, 2019[15]). Low thrombin concentrations exert neuroprotective effects whereas increased thrombin concentrations lead to neurotoxic consequences with edema formation and inflammation, which should be countered by endogenous brain thrombin inhibitors. Thrombin could be activated by chronic inflammation or tissue injury and again facilitate inflammatory responses by secretion of inflammatory cytokines influencing either astrocytes/microglia growth, migration or proliferation, and even angiogenesis (Gofrit and Shavit-Stein, 2019[15]). Glioma cells in culture produced thrombin that in turn increased their proliferation and adhesion to matrix proteins (Gofrit and Shavit-Stein, 2019[15]). The cancer microenvironment and systemic homeostasis, including the coagulation system, is a part of the vascular milieu that remains under the control of prototypic oncogenic pathways (Magnus et al., 2014[36]). Specific phenotypes of cancer cells may influence the state of systemic coagulation and brain cancer is one of those with a greater capacity to stimulate the coagulation system.

The increase of ATIII in our patients with glioma could indicate an attempt to counterbalance an overproduction of thrombin following the inflammatory-thrombotic processes however without having any predictive value for the overall survival in this study. Indeed, our data should be considered preliminary because of some study shortcomings firstly due to its retrospective design and the small sample size but mainly related to population heterogeneity regarding the type of tumor and the variable disease stage at study inclusion. Nevertheless, the evaluation of routine laboratory parameters such as NLR, ATIII, FVIII and d-dimer in this setting could represent a real-life picture of disease progression.

## Conclusions

Our study showed higher levels of either NLR, ATIII, FVIII or d-dimer in glioma patients compared to both MS patients and healthy controls indicating the first three indicators as moderately accurate biomarkers for this cancer. Among these markers, only NLR was found to be predictive of OS even though it was not, in this case, a sufficiently accurate biomarker. These findings could indicate the presence of increased inflammatory-thrombotic processes most likely due to augmented innate immunity at the expense of the adaptive immunity in neoplastic disease such as glioma patients compared to healthy controls but even to an inflammatory disorder like MS. Also, the predictive value of steroid therapy for the overall survival of patients with glioma, which is known to specifically reduce adaptive immunity, reinforces this hypothesis. A better understanding of this complex link may lead to new diagnostic and therapeutic perspectives for glioma patients in terms of strengthening their adaptive immunity in addition to the best and more adequate anti-cancer therapies. 

## Funding

This research received no external funding.

## Acknowledgements

We thank Dr Silvana Zannino for her assistance with the database used in this work.

## Institutional review board statement

The study was conducted according to the guidelines of the Declaration of Helsinki, and approved by the Institutional Ethics Committee of IRCCS Regina Elena National Cancer Institute (protocol code 0002720, date of approval 02-24-2020).

## Informed consent statement

Informed consent was obtained from all subjects involved in the study.

## Data availability statement

Publicly available datasets were analyzed in this study (Raw data). 

## Conflict of interest

The authors declare that they have no conflict of interest.

## Supplementary Material

Supplementary data

## Figures and Tables

**Table 1:**
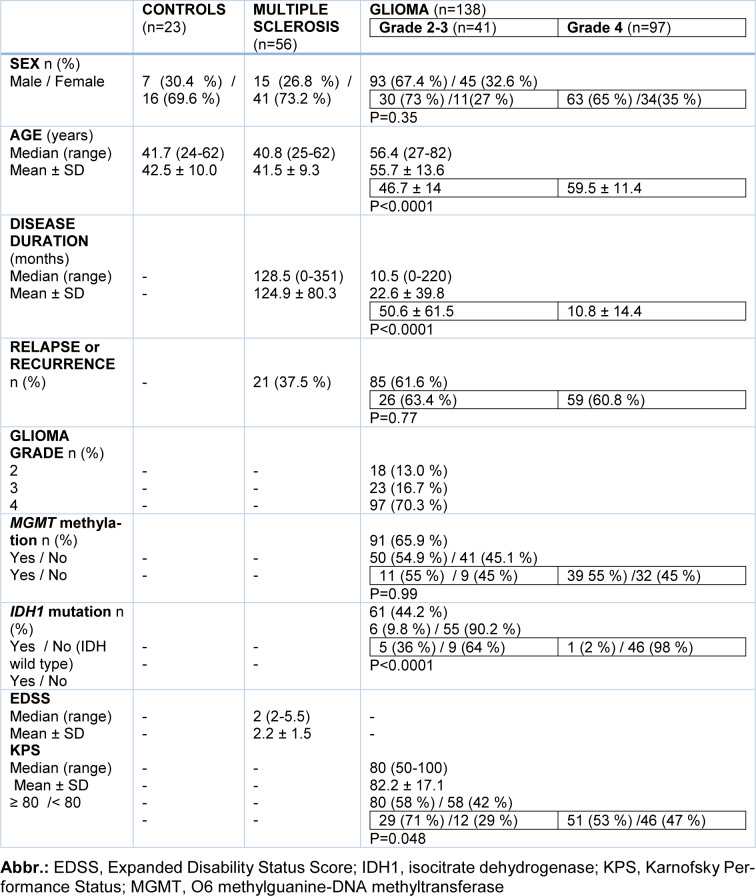
Demographic and clinical characteristics of study participants

**Table 2:**
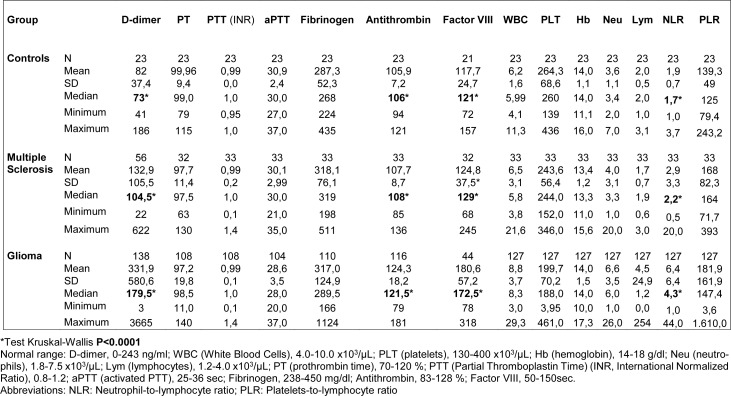
Coagulation factors and complete blood count in controls, multiple sclerosis and glioma patients

**Table 3:**
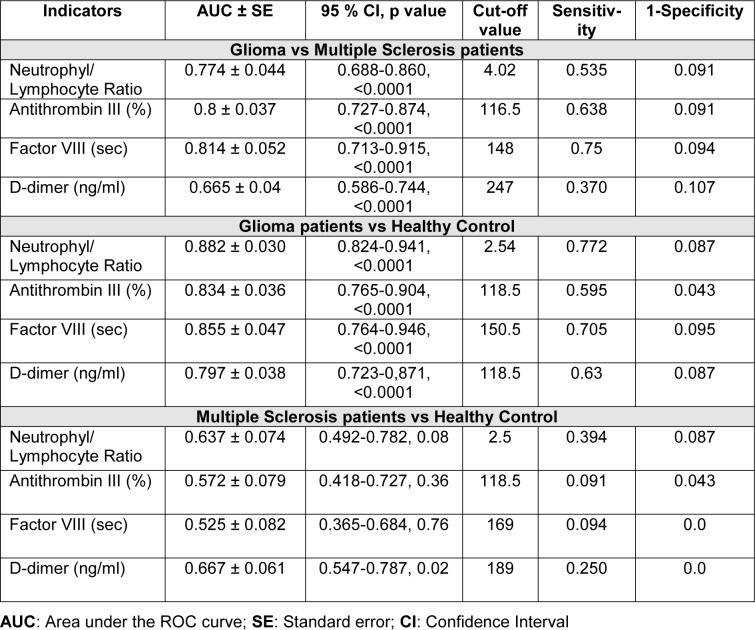
ROC curve analysis and cutoff values for Neutrophyl/Lymphocyte ratio and coagulation factors (Antithrombin III, Factor VIII, d-dimer) in glioma patients versus multiple sclerosis patients or healthy controls

**Table 4:**
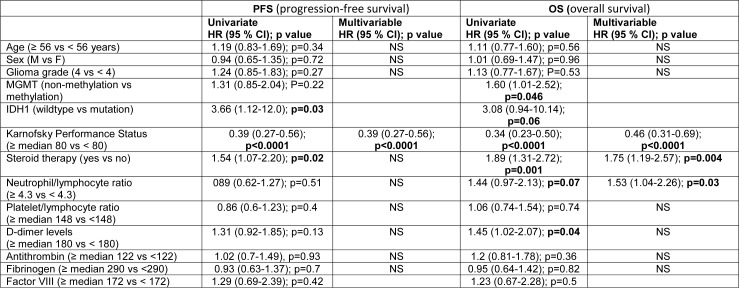
Univariate and multivariable analyses for Progression Free Survival and Overall Survival in glioma patients
